# Searching for Controlled Trials of Complementary and Alternative Medicine: A Comparison of 15 Databases

**DOI:** 10.1093/ecam/nep038

**Published:** 2011-06-23

**Authors:** Elise Cogo, Margaret Sampson, Isola Ajiferuke, Eric Manheimer, Kaitryn Campbell, Raymond Daniel, David Moher

**Affiliations:** ^1^Children's Hospital of Eastern Ontario Research Institute, Ottawa, Canada; ^2^Faculty of Information and Media Studies, University of Western Ontario, London, Canada; ^3^Department of Information Studies, University of Wales, Aberystwyth, UK; ^4^Center for Integrative Medicine, University of Maryland School of Medicine, Baltimore, MD, USA; ^5^Program for Assessment of Technology in Health, McMaster University, St Joseph's Healthcare, Hamilton, Canada; ^6^Clinical Epidemiology Program, Ottawa Health Research Institute, Canada; ^7^Department of Epidemiology and Community Medicine, Faculty of Medicine, University of Ottawa, Ottawa, Canada

## Abstract

This project aims to assess the utility of bibliographic databases beyond the three major ones (MEDLINE, EMBASE and Cochrane CENTRAL) for finding controlled trials of complementary and alternative medicine (CAM). Fifteen databases were searched to identify controlled clinical trials (CCTs) of CAM not also indexed in MEDLINE. Searches were conducted in May 2006 using the revised Cochrane highly sensitive search strategy (HSSS) and the PubMed CAM Subset. Yield of CAM trials per 100 records was determined, and databases were compared over a standardized period (2005). The Acudoc2 RCT, Acubriefs, Index to Chiropractic Literature (ICL) and Hom-Inform databases had the highest concentrations of non-MEDLINE records, with more than 100 non-MEDLINE records per 500. Other productive databases had ratios between 500 and 1500 records to 100 non-MEDLINE records—these were AMED, MANTIS, PsycINFO, CINAHL, Global Health and Alt HealthWatch. Five databases were found to be unproductive: AGRICOLA, CAIRSS, Datadiwan, Herb Research Foundation and IBIDS. Acudoc2 RCT yielded 100 CAM trials in the most recent 100 records screened. Acubriefs, AMED, Hom-Inform, MANTIS, PsycINFO and CINAHL had more than 25 CAM trials per 100 records screened. Global Health, ICL and Alt HealthWatch were below 25 in yield. There were 255 non-MEDLINE trials from eight databases in 2005, with only 10% indexed in more than one database. Yield varied greatly between databases; the most productive databases from both sampling methods were Acubriefs, Acudoc2 RCT, AMED and CINAHL. Low overlap between databases indicates comprehensive CAM literature searches will require multiple databases.

## 1. Introduction

The continuing growth in usage and popularity of complementary and alternative medicine (CAM) [1, 2] has resulted in an increase in scientific research, including systematic reviews, in this field [3, 4]. Some hierarchies of evidence suggest that high-quality systematic reviews of randomized controlled trials (RCTs) are the gold standard of evidence-based medicine since they attempt to synthesize research using a rigorous methodology to limit bias [5, 6]. Part of this methodology includes identifying a comprehensive evidence base of controlled clinical trials (CCTs—including randomized controlled trials) through a highly sensitive search of the literature [7]. Such broad searches have resource implications for the conduct of systematic reviews, so knowledge of various bibliographic databases' (db) productivity, overlap and features is important. In addition, practitioners, researchers and librarians searching for high-quality evidence of CAM may not know which or how many databases to search in order to obtain a totality of evidence.

The objective of this project is to compare 15 bibliographic databases relevant to CAM in order to ascertain the unique contributions of each; as well as a study of other features of these databases, such as searchability and cost. Such a broad comparison of CAM databases has not been undertaken to date. A literature review of similar research provided the findings summarized below.

A recent study by Pilkington (2007) analysed original sources of controlled trials included in 35 CAM systematic reviews and then determined whether the 127 trials identified were indexed in MEDLINE, EMBASE, Cochrane CENTRAL (Cochrane Controlled Trials Register or CCTR) and three other databases (AMED, CINAHL and PsycINFO) [8]. The study found that 27 (out of the 127) trials were uniquely found in one of the databases and AMED produced the highest number of unique citations (*n* = 5) [8]. Sampson et al. [3] analysed 13 databases for pediatric CAM RCTs, identifying over 900 relevant records from over 300 journals. The study concluded that for an exhaustive search for pediatric CAM RCTs, CAB Health, CINAHL and AMED should also be searched in addition to MEDLINE, EMBASE and CENTRAL (CCTR) [3]. Murphy et al. [9] analysed 13 databases for trials to be included in a systematic review on the reliability of spinal palpation. The study found that of the 49 trials (retrieved from the databases) that were determined to be relevant for the systematic review, MANTIS was the most productive database (*n* = 35), followed by PubMed, CINAHL and MD Consult (*n* = 19 each) [9].

Sood et al. [10] analysed databases used in 10 acupuncture systematic reviews. The study found that PubMed indexed 69% of the total 108 included acupuncture trials and the number of databases searched varied considerably from 3 to 12 (median 5) [10]. Vickers [11] analysed The Cochrane Collaboration Complementary Medicine Field's registry of 3774 complementary medicine RCTs from 965 journals. The study found that 81% of the RCTs were indexed in MEDLINE and only about one-third of these could easily be found by a MEDLINE search [11]. Aker et al. [12] studied three databases for chiropractic literature, identifying 385 included citations. The study found that MEDLINE retrieved 68% of the citations, CHIROLARS retrieved 23% and Index to Chiropractic Literature retrieved 10% [12]. McPartland and Pruitt [13] conducted an extensive literature search on a herbal medicine topic (saw palmetto) that included searching MEDLINE and four additional databases (EMBASE, Cochrane, AGRICOLA and IBIS) along with hand searching of non-indexed herbal journals. They found that MEDLINE yielded only 33% of the 58 relevant clinical trials identified [13].

Moher et al. [14] analysed the reporting in pediatric CAM systematic reviews, and found that they were ‘particularly weak in terms of the comprehensiveness in their search to identify primary studies', (page 9) with only 40% reporting a search that was reasonably comprehensive. Shekelle et al. [15] analyzed the sources and methods used to identify CAM evidence in 21 Evidence-based Practice Center reports on CAM interventions. The study concluded that CAM systematic reviewers should include CAM specialized databases in their search methods [15]. Additional prior research comparing databases and analyzing the bibliometrics of CAM literature has either studied a specific area of CAM or focused on a small number of databases [16–26].

As can be seen from the literature review above, a broad comparison of CAM databases has not been undertaken to date. This project studies 15 databases beyond the three major databases (MEDLINE, EMBASE, and CENTRAL/CCTR) in an attempt to identify which additional databases might be useful sources of CAM controlled trials. The results of this project may benefit systematic reviewers, researchers, practitioners and librarians involved with CAM.

This project was part of a larger program to update The Cochrane Collaboration Complementary Medicine Field's trials registry, which is a database containing information on CAM controlled clinical trials. The definition of CAM used in this study was based on the one provided by the National Center for Complementary and Alternative Medicine (NCCAM), which defines CAM as health care practices and products that are “not presently considered to be part of conventional medicine” [27]. Examples of the CAM therapies and modalities covered in this study include, *but are not limited to*: acupuncture, chiropractic, hands-on healing, herbal medicine, homeopathy, hypnosis, magnetotherapy, massage therapy, mind-body techniques, naturopathic medicine, osteopathic manipulation, prayer, yoga, etc. Nutritional supplements and diet therapy were considered to be CAM if they are not used in conventional medicine; for example, high-dose B-vitamin therapy was considered to be CAM, whereas a (routine-dose) prenatal multivitamin was not considered to be CAM.

## 2. Methods

**Figure 1:**
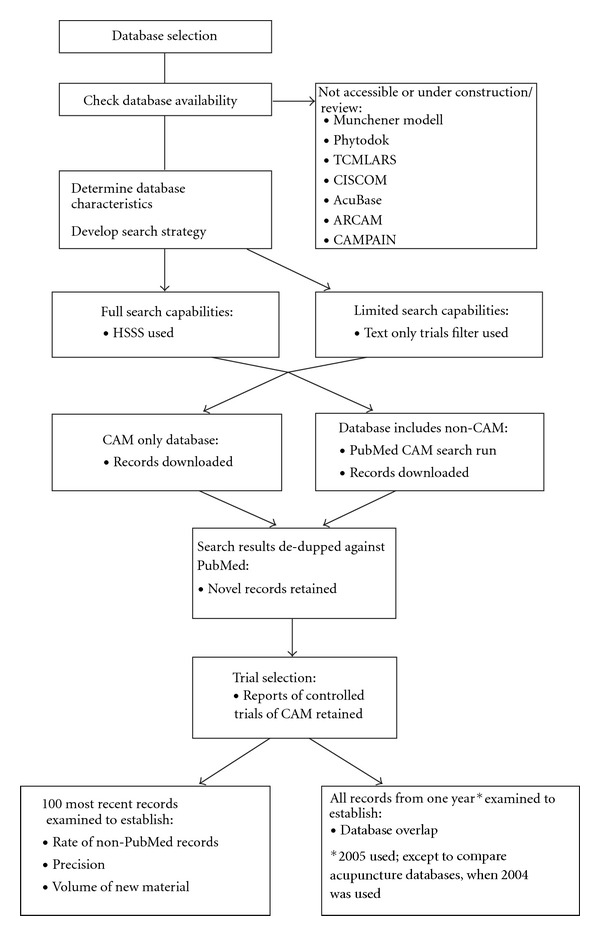
Flow chart of study methods.

Figure 1 summarizes the sequence of the study methods used. Further details are provided below.

### 2.1. Database Selection and Availability

Databases other than MEDLINE, CENTRAL and EMBASE were selected as potentially useful sources of CAM controlled trials from lists of resources provided on the websites of the Research Council for Complementary Medicine (available at http://www.rccm.org.uk/static/Links_CAM_databases.aspx?m=11) and others. Databases were selected for analysis if they contained primary reports of clinical trials (versus only topic summaries, consumer information, reviews, etc.) and they included CAM therapies. Cost was not a factor in database selection, and the only language restriction was the exclusion of Chinese (since this was allocated to another research team).

Twenty-two databases were chosen for study consideration based on their likely potential to be highly productive sources of CAM controlled trials. Seven of these were then excluded since we were unsuccessful in obtaining access to the Munchener Modell, Phytodok and TCMLARS databases, and the CISCOM, AcuBase, ARCAM and CAMPAIN databases were under construction/review at the time of this study and were also unavailable. The following 15 databases were included: Acubriefs, Acudoc2 RCT (ECR), AGRICOLA, Alt HealthWatch, AMED, CAIRSS, CINAHL, Datadiwan, Global Health, Herb Research Foundation, Hom-Inform, IBIDS, Index to the Chiropractic Literature, MANTIS and PsycINFO. A brief description of each database selected is presented in Appendix S1 available online as Supplementary Data. A complete list of the abbreviations used in this report is provided in the appendix.

### 2.2. Database Characteristics and Search Strategy Development

Information on database features, the number of journals covered by each database, database costs and a description of each database were obtained by the database producer/vendor or determined by the authors through exploration.

The search strategy used in CAM-specific databases to retrieve controlled trials was the 2006 revised Cochrane highly sensitive search strategy (HSSS) or a modified version of it, depending on the functionality of the search interface [28]. This evidence-based search strategy has been developed to retrieve a very high number of controlled trials in MEDLINE. For databases that are not CAM-specific, the PubMed CAM Subset search strategy was added into the search, or a modified version of it, depending on the functionality of the search interface. The PubMed CAM Subset search strategy was introduced in 2003, and is available at http://www.nlm.nih.gov/bsd/pubmed_subsets/comp_med_strategy.html. The Ovid interface translation of the CAM Subset is available at http://www.compmed.umm.edu/integrative/cochrane_ovid.asp. (The search strategies used are available upon request from the authors.)

Some of the databases studied do not have a thesaurus or any indexing, so we developed several text-word versions of the revised HSSS and tested them against records entering PubMed in 2000, a year for which the Cochrane-National Library of Medicine (NLM) re-tagging initiative (of controlled trials) has been completed. We selected the one giving the highest recall of RCTs from all of PubMed and the CAM Subset. The search strategy selected was: (placebo or randomized or randomised or randomly or (random$ adj2 allocat$) or (clinical adj2 trial)).tw. Any relevant index terms available in the database being searched were added using the Boolean OR operator.

### 2.3. De-Duplication against PubMed

The searches were carried out in May 2006. Overlap with PubMed was removed using the PubMed Batch Citation Matcher technique developed by Sampson to eliminate records from journals indexed in PubMed and then further de-duplicated (between all the database non-PubMed retrievals) manually in Reference Manager [29]. (The Reference Manager import filter styles are available upon request from the authors.) We were unable to import the Acudoc2 RCT and Hom-Inform records into Reference Manager and so those databases had to be screened online.

### 2.4. Trial Selection

The eligibility criteria were for an item to be both CAM related and a completed controlled trial (RCT or CCT). The definition of CAM used in this study is provided in the Introduction above. Vickers (1998) discussed the “grey area” that exists between CAM and conventional medicine [11]. Our research team had CAM expertise, and screening decisions regarding CAM criteria erred on the side of inclusion. Records found to be relevant were checked for inclusion in all the other databases under consideration, as well.

### 2.5. Data Analysis

Two samples from each database were selected after de-duplicating against PubMed, and then screened for eligibility: (i) the 100 most recent records, and then (ii) all from the year 2005. Standardizing the time frame permitted examination of overlapping coverage.

The formulae below were used to analyze the results of the data obtained after de-duplication of the database retrievals against PubMed:

(1) $$\begin{array}{cc} \text{Coverage} & =\frac{\begin{array}{c} \text{No}.\;\text{of}\;\text{controlled}\;\text{CAM}\;\text{trials}\;\text{included}\;\text{in}\;\text{a}\;\text{database}\times 100\text{\%} \end{array}}{\begin{array}{c} \text{Total}\;\text{no}:\;\text{of}\;\text{controlled}\;\text{CAM}\;\text{trials}\;\text{included}\;\text{in}\;\text{all}\;15\;\text{databases} \end{array}},\\ \text{Precision} & =\frac{\begin{array}{c} \text{No}.\;\text{of}\;\text{controlled}\;\text{CAM}\;\text{trials}\;\text{included}\;\text{in}\;\text{a}\;\text{database}\;\left(\text{db}\right)\times 100\text{\%} \end{array}}{\begin{array}{c} \text{No}.\;\text{of}\;\text{trials}\;\text{screened}\;\text{from}\;\text{the}\;\text{database} \end{array}},\\ \text{Database}\;\text{overlap} & =\frac{\begin{array}{c} \text{No}.\;\text{of}\;\text{controlled}\;\text{CAM}\;\text{trials}\;\text{included}\;\text{in}\;\text{row}\;\text{db}\;\text{that}\;\text{were}\;\text{also}\;\text{found}\;\text{in}\;\text{column}\;\text{db} \end{array}}{\begin{array}{c} \text{No}.\;\text{of}\;\text{controlled}\;\text{CAM}\;\text{trials}\;\text{included}\;\text{in}\;\text{row}\;\text{database}\;\left(\text{db}\right) \end{array}}. \end{array}$$

It was noted that the Acubriefs pilot screening set of 100 most recent records only went as far back as 2005 and Acudoc2 RCT did not have any records after 2004. Since a direct comparison of the two acupuncture-specific databases in the study was determined to be useful, a separate analysis of these two databases was conducted with relevant articles published in 2004 (since the two main sample analyses mentioned above could not capture a comparison between the acupuncture databases).

## 3. Results

### 3.1. Database Productivity

Table 1 indicates the number of journals indexed in the 15 databases and the number of records that had to be examined in order to obtain 100 records not already included in PubMed. The retrieval size required to be downloaded and de-duplicated to eliminate records from journals indexed in PubMed indicates the marginal yield beyond what can be identified by searching PubMed. For example, 1520 records from AMED had to be downloaded and de-duplicated against PubMed in order to get 100 non-PubMed records for screening; whereas, only 160 records from Acudoc2 RCT had to be de-duplicated against PubMed in order to get 100 non-PubMed records for screening.

Precision was then determined on the basis of the 100 most recent records. The time period covered varies by database (Table 1). After de-duplication between all of the databases, a total of 305 trials were included across the 10 databases that were found to be productive (after de-duplication against PubMed).

Five of the 15 databases studied were found to be unproductive from the pilot, and were not analyzed further in the comparisons. These were: AGRICOLA, CAIRSS, Datadiwan, Herb Research Foundation and IBIDS. AGRICOLA yielded no eligible records. CAIRSS produced one eligible record. Datadiwan did not retrieve any controlled trials from the large sample that was screened online. Herb Research Foundation retrieved only 111 records for the entire database before checking journals against PubMed. All material retrieved from IBIDS was from journals indexed in PubMed.

Acudoc2 RCT had the highest precision with all 100 trials being included. Databases focusing on certain CAM practices, such as acupuncture (Acudoc2 RCT and Acubriefs), homeopathy (Hom-Inform) or chiropractic (ICL), were intermixed with CAM databases with broader coverage (AMED, Alt HealthWatch) and those covering CAM and non-CAM practices (such as CINAHL and PsycINFO).

Most databases yielded 100 eligible articles since 2004, although Acudoc2 RCT had no recent records (most recent was from 2004) and Hom-Inform produced 30 included trials in the 100 records over a 6-year span (with the most recent being from 2003).

### 3.2. Database Overlap

Standardizing the time frame permits examination of overlapping coverage; Table 2 summarizes the analysis of the eight relevant sources from 2005. After de-duplication between all of the databases, a total of 255 eligible articles were identified from 2005 (after de-duplication against PubMed). Acubriefs and CINAHL were by far the most productive databases with 152 and 62 included trials found, covering 60% and 24% of all relevant trials found, respectively. AMED was third in terms of overall productivity with 25 included trials (10% of the total). Global Health, PsycINFO and MANTIS had between 10–17 trials, (4–7% of the total). Alt HealthWatch (AHW) was found to be the least productive database, followed by ICL, with 3 and 4 trials found, for coverage of 1% and 2%, respectively.

The number of unique trials found in each database (Table 2) was high for the two most productive databases, Acubriefs and CINAHL (138 and 48, resp.), but was relatively lower for the third most productive database overall, AMED. AMED placed fifth in terms of number (*N*) of unique trials (*N* = 7) since over two-thirds of its included trials were found in other databases. Global Health and PsycINFO produced the third and fourth highest number of unique trials (*N* = 15 and *N* = 14), respectively. MANTIS and Alt HealthWatch each produced 3 unique trials, while ICL produced 1 unique trial.

Only 10% of all included trials were found in more than one database and most of these (*N* = 20, or 8%) were in two databases, four (2%) were in three databases and two (1%) were found four databases.  Table 3 shows the overlap between pairs of databases in 2005 for included trials after de-duplication against PubMed. ICL and MANTIS had the highest overlap with each of the other databases. ICL and MANTIS had trials overlapping in CINAHL (50% each), AMED (50% each) and with each other. CINAHL was the database that accounted for the largest portion of the overlapping trials found in the other databases. MANTIS also had some overlap with Global Health and with PsycINFO (10% each). AMED had the third highest percentage of its included trials that overlapped in each of the other databases, most notably with Acubriefs (36%), CINAHL (28%) and MANTIS (20%). The three trials found in Alt HealthWatch were all unique, so there was no overlap for this database. Acubriefs, CINAHL, Global Health and PsycINFO all had relatively low overlap with each of the other databases.

As with the analysis to determine precision, overlap between the two databases that are specific to acupuncture and traditional Chinese medicine (TCM) was examined by comparing an older time frame (2004). Twenty-one of these Acudoc2 RCTs were found to be overlapping in Acubriefs, and 69 were unique. This gives an overlap of 23% for Acudoc2 RCT trials also found in Acubriefs.

Comparing the 2005 analysis (Table 2) with the most recent 100 records (Table 1), Acubriefs and AMED were similarly in the top 3 for productivity, and Alt HealthWatch and ICL were similarly found to be the least productive overall.

### 3.3. Database Features

Table 4 gives cost information for the 15 databases. Eight of them are free, and two are available for less than US$200. Table 4 also provides details of the search interface features of the 15 databases studied. Note that some databases do not allow for sophisticated or long search strategies, in particular Acudoc2 RCT, Datadiwan and Herb Research Foundation.

## 4. Discussion

### 4.1. Major Findings

From the 15 databases studied, five were found to be unproductive from the pilot screening of the 100 most recent records. These were: AGRICOLA, CAIRSS, Datadiwan, Herb Research Foundation and IBIDS. Sampson et al. [3] found that AGRICOLA and IBIDS did not produce any unique coverage of pediatric CAM RCTs (although the databases chosen for that analysis were not all the same as those in the present study). Systematic reviewers may prefer to search MEDLINE instead of databases without unique coverage since MEDLINE offers better indexing and more search features.

The most striking finding of this study is the remarkably low level of overlap that was found between the databases once overlap with PubMed was removed. Only 10% were indexed in more than one of the databases studied, and most of these were found in only two databases.

The overlap between each pair of databases studied from the 2005 analysis found that CINAHL was the database that accounted for the largest portion of the overlapping trials found in the other databases, that is, searching CINAHL would retrieve more of the overlapping trials found in the other databases than searching any other single database studied.

Two of the databases were found to be out of date (Acudoc2 RCT and Hom-Inform). Reviewers working with specialty databases need to test currency, and may wish to supplement their database searching with targeted hand searching of recent issues of journals not indexed in databases that are up to date.

### 4.2. Future Research Areas

Although this study did not remove duplicates against EMBASE or CENTRAL (CCTR) and therefore the number of included trials that would have been found in those databases is not known, we believe that a substantial proportion of them will not be identified in EMBASE or CENTRAL for two reasons. First, there is overall a relatively high degree of overlap between MEDLINE and these 2 databases [30], and the trials found in this study were from journals not indexed in PubMed. Second, the pediatric CAM RCT study by Sampson et al. [3] found that three of the databases studied (CAB Health, CINAHL and AMED) produced unique RCTs that were not in MEDLINE, EMBASE or CCTR (CENTRAL). Some additional support for this is obtained from the study by Pilkington, which found that none of the six databases studied listed all 127 included trials and all of the databases except MEDLINE listed at least one unique trial [8].

This study did not analyze the included trials by type of CAM/therapeutic modality (e.g., acupuncture, chiropractic, herbal medicine, nutritional supplementation, spiritual healing, etc.) but given the breadth of the CAM field, it is possible that certain modalities may have more overlap in terms of both databases and journals which could potentially decrease the number of databases that should be searched for a systematic review that focuses on a specific type of CAM. We found some overlap between the two acupuncture databases and between the chiropractic and manual therapy databases. However, from the findings of this study, a systematic review that includes all types of CAM (e.g., a review of CAM interventions for menopause) may benefit from searching as many of the productive databases as possible in addition to MEDLINE (PubMed). In addition, it may be beneficial to search as many databases as possible if they cover the type of CAM being reviewed (see Appendix S1 available as Supplementary Data for database descriptions). It is interesting to note that the study by Pilkington, which analyzed 6 databases based on the type of CAM therapy, also concluded that it is important to search a range of databases when conducting a CAM systematic review [8].

### 4.3. Top Databases

Of the six databases covering a broader range of CAM (i.e., not focused on specific modalities), CINAHL was the most productive, followed by AMED. Acubriefs and Acudoc2 RCT databases were highly productive for acupuncture trials. The large proportion of trials from acupuncture databases that were included from journals not indexed in PubMed is notable. In contrast, an analysis by Raschetti et al. [16] of MEDLINE publications on “complementary therapies” from 1997 to 2002 found that of the over 1500 CAM RCTs identified, 17% were on acupuncture (and 28% on phytotherapy, 12% on manipulative practices, and 3% on homeopathy). One hypothesis is that there may be a large number of Chinese journals that publish acupuncture controlled trials that are not indexed in PubMed. It is not surprising that the Acudoc2 RCT database had the highest precision since only RCTs and CAM topics (acupuncture and traditional Chinese medicine) are included in it.

### 4.4. Technical Challenges

Including multiple databases in a systematic review has resource implications beyond cost. Acudoc2 RCT and Hom-Inform had to be screened online and duplicates identified manually (in MS Word) since it was too difficult to import the records into Reference Manager. The necessity for online screening is a significant draw-back to systematic reviewers as the searching is often undertaken by a librarian and screening by clinicians. In addition, several of the databases had large proportions of their retrievals from journals indexed in PubMed. The time required to download and de-duplicate PubMed material would have been much greater if the technique developed by Sampson to eliminate records from journals indexed in PubMed had not been used [28]. Therefore, removing PubMed overlap significantly decreased the screening burden and eliminated redundancy.

### 4.5. Database Features

The study of database features indicates that some of the search interfaces do not allow for complex searches, in particular Acudoc2 RCT and to some degree also Hom-Inform. In contrast, the other acupuncture database studied, Acubriefs, has several advanced searching features. Cost is a factor in database access for some systematic reviewers, librarians and practitioners. Regarding the costs to access the 10 productive databases, half of them are either available for free (*n* = 3) or for less than US$200 (*n* = 2). Some of the more expensive databases are accessible to those with institutional access, such as through a university or hospital, but few universities subscribe to all of the 15 databases studied.

## 5. Conclusions

Examination of the portion of CAM databases from sources not also indexed in PubMed narrowed the field from 15 candidates to 10 more productive sources. The very low overlap between these non-PubMed sources suggests the need for multiple database searching in addition to MEDLINE in order to comprehensively search for CAM controlled trials. The results indicate that of the six databases analyzed that are not focused on a specific therapy, CINAHL was the most productive, followed by AMED. The Acubriefs and Acudoc2 RCT databases were highly productive for acupuncture trials. Databases not considered in this survey can be rapidly assessed by considering currency and removing the overlap with PubMed using batch processes.

## Supplementary Data

Supplementary data are available at *eCAM* Online.

## Supplementary Material

A brief description of the following 15 databases: Acubriefs, Acudoc2 RCT (ECR), AGRICOLA, Alt HealthWatch, AMED, CAIRSS, CINAHL, Datadiwan, Global Health, Herb Research Foundation, Hom-Inform, IBIDS, Index to the Chiropractic Literature, MANTIS and PsycINFO.

## Figures and Tables

**Table 1:** Database productivity from 100 most recent non-PubMed records (before screening).

	*N* of journals indexed	Estimated *N* of non-PubMed journals indexed	*N* examined to get 100 non-PubMed records	Eligible per 100 (precision, %)	Timeframe for 100 non-PubMed records
Acudoc2 RCT	n/a	n/a	160	100	2003–2004
Acubriefs	32	20	337	56	2005+
AMED	508	230	1520	41	2004+
Hom-Inform	53	43	373	30	1998–2003
MANTIS	1541	565	1082	29	2004+
PsycINFO	2056	1147	1015	27	2004+
CINAHL	1827	855	1490	25	2005+
Global Health	5701	3362	1303	19	2004+
ICL	44	39	221	14	2004+
Alt HealthWatch^a^	35	21	531	9	2004+
CAIRSS for Music	18	15	n/a	1	n/a
AGRICOLA	2241	1593	986	0	n/a
Datadiwan	n/a	n/a	n/a	0	n/a
Herb Research Foundation	n/a	n/a	n/a	0	n/a
IBIDS	5257	1208	n/a	0	n/a

*N* indicates number; n/a indicates information not available.

^a^We examined the peer-reviewed journals (PRJ subset) only.

**Table 2:** Summary of relevant articles published in 2005.

Database	Acubriefs	CINAHL	AMED	Global Health	PsycINFO	MANTIS	ICL	AHW
*N* included	152	62	25	17	16	10	4	3
*N* unique	138	48	7	15	14	3	1	3
*N* overlap	14	14	18	2	2	7	3	0
Coverage^a^ (%)	60	24	10	7	6	4	2	1

^a^Coverage indicates the percentage of total relevant 2005 articles identified (*N* = 255) that were retrieved from that source.

**Table 3:** Database overlap for 2005.

	Acubriefs	CINAHL	AMED	Global Health	PsycINFO	MANTIS	ICL	AHW
Acubriefs	1.00	0.03	0.06	0.00	0.00	0.00	0.00	0.00
CINAHL	0.08	1.00	0.11	0.02	0.02	0.08	0.03	0.00
AMED	0.36	0.28	1.00	0.04	0.04	0.20	0.08	0.00
Global Health	0.00	0.06	0.06	1.00	0.00	0.06	0.00	0.00
PsycINFO	0.00	0.06	0.06	0.00	1.00	0.06	0.00	0.00
MANTIS	0.00	0.50	0.50	0.10	0.10	1.00	0.30	0.00
ICL	0.00	0.50	0.50	0.00	0.00	0.75	1.00	0.00
AHW	0.00	0.00	0.00	0.00	0.00	0.00	0.00	1.00

This table shows the proportion of controlled CAM trials (after de-duplication against PubMed) from the database listed in the row that were also indexed in the database shown in the column.

**Table 4:** Database costs and search interface features.

	Cost	Truncation	Adjacency	Combine sets	Thesaurus or index	Limits	Phrase searching
Acubriefs	<$200	Yes	No	Yes	Yes	Yes	No
Acudoc2 RCT	Free	Yes	No	No	No	No	No
AGRICOLA	Free	Yes	No	Yes	Yes	Yes	Yes
Alt HealthWatch	>$200	Yes	Yes	Yes	Yes	Yes	Yes
AMED	>$200	Yes	Yes	Yes	Yes	Yes	Yes
CAIRSS for Music	Free	Yes	No	Yes	No	Yes	Yes
CINAHL	>$200	Yes	Yes	Yes	Yes	Yes	Yes
Datadiwan	Free	Yes	No	No	No	No	Yes
Global Health	>$200	Yes	Yes	Yes	Yes	Yes	Yes
Herb Research Foundation	Free	Yes	No	No	No	Yes	Yes
Hom-Inform	Free	Yes	No	No	Yes	Yes	No
IBIDS	Free	Yes	No	Yes	Yes	Yes	Yes
ICL	Free	Yes	No	Yes	Yes	Yes	Yes
MANTIS	<$200 through Health Index	Yes	Yes	Yes	Yes	Yes	Yes
PsycINFO	>$200	Yes	Yes	Yes	Yes	Yes	Yes

## Appendix

### List of Abbreviations (and Long Forms of Database Names)

AGRICOLA: Agricultural Online Access

AHW: Alt HealthWatch

AMED: Allied and Complementary Medicine

ARCAM: Arthritis and Complementary Medicine

CAIRSS: Computer-Assisted Information Retrieval Service System

CAM: complementary and alternative medicine

CAMPAIN: Complementary and Alternative Medicine and Pain

CCT: controlled clinical trial

CCTR/CENTRAL: Cochrane Controlled Trials Register

CINAHL: Cumulative Index to Nursing and Allied Health

CISCOM: Centralised Information Service for Complementary Medicine

CM: complementary medicine

Db: database

GERA: Groupe d'Études et de Recherches en Acupuncture [Group of Studies and Research in Acupuncture]

HSSS: highly sensitive search strategy

IBIDS: International Bibliographic Information on Dietary Supplements

ICL: Index to the Chiropractic Literature

MANTIS: Manual, Alternative and Natural Therapy Index System

*N*: number

NCCAM: National Center for Complementary and Alternative Medicine

NIH: National Institutes of Health

PRJ: peer-reviewed journals

RCT: randomized controlled trial

TCM: traditional Chinese medicine

TCMLARS: Traditional Chinese Medical Literature Analysis and Retrieval System.
