# Supplementary material for: Searching for Controlled Trials of Complementary and Alternative Medicine: A Comparison of 15 Databases
Source: Evid Based Complement Alternat Med. 2011 Jun 23;2011:858246. doi: 10.1093/ecam/nep038 (PMC3137728; doi:10.1093/ecam/nep038)
Supplement: Supplementary file 1 — A brief description of the following 15 databases: Acubriefs, Acudoc2 RCT (ECR), AGRICOLA, Alt HealthWatch, AMED, CAIRSS, CINAHL, Datadiwan, Global Health, Herb Research Foundation, Hom-Inform, IBIDS, Index to the Chiropractic Literature, MANTIS and PsycINFO. [file 858246.f1.pdf]

Appendix 1 - Database descriptions

Acubriefs - By the Best of Both Worlds Foundation and the Medical Acupuncture Research Foundation (MARF). “Its purpose is to make available the most comprehensive database of references on acupuncture in the English language”.
http://www.acubriefs.com/

Acudoc2 RCT - The English version of a bibliographic database of acupuncture RCTs by GERA.
http://www.acupuncture-medicale.org/ecracu/section1a.html

AGRICOLA - The National Agricultural Library's two databases that index the books and articles of the agricultural research literature, including plant sciences and nutrition.
http://agricola.nal.usda.gov/

Alt HealthWatch – An alternative medicine database that offers full-text articles from more than 170 international peer-reviewed and professional journals, magazines, and other sources. Available through EBSCO.
http://www.epnet.com/

AMED - By the British Library. An English language database of allied and complementary medicine. It contains bibliographic data, keywords, and abstracts since 1985 from journals in complementary medicine, palliative care, and several professions allied to medicine. (This project used it via a subscription with OVID).
http://www.bl.uk/collections/health/amed.html

CAIRSS for Music - By the University of Texas at San Antonio. A bibliographic database of research literature in music education, music psychology, music therapy, and music medicine.

http://imr.utsa.edu/CAIRSS.htm

CINAHL - A bibliographic database of nursing literature, as well as materials from 17 allied health disciplines, plus biomedicine, management, behavioural sciences, health sciences librarianship, education, and consumer health. (This project used it via a subscription with OVID).

http://www.cinahl.com/prodsvcs/cinahldb.htm

Datadiwan – A German holistic medicine database including research articles, some in English. By Patienteninformation für Naturheilkunde.

http://www.datadiwan.de/suche/index_e.htm

Global Health (formerly CAB Health) - A public health database that provides information on international health, biomedical life sciences, non-communicable diseases, public health nutrition, food safety and hygiene, and much more. Records date back to 1973. Available through EBSCO.

http://www.cabi.org/datapage.asp?iDocID=169

Herb Research Foundation – A website of herbal research information provided by the Herb Research Foundation.

http://www.herbs.org/index.html

Hom-Inform – A database of literature references of homoeopathy, with key terms and some abstracts, provided by the British Homeopathic Library.

http://www.hom-inform.org/

IBIDS - By the Office of Dietary Supplements at the National Institutes of Health. It is a database of published, international, scientific literature on dietary supplements, including vitamins, minerals, and botanicals.
http://dietary-supplements.info.nih.gov/Health_Information/IBIDS.aspx

Index to Chiropractic Literature (ICL) - By the Chiropractic Library Consortium. A database of literature published in selected chiropractic journals, from 1985 to the present for most journals.
http://www.chiroindex.org/

MANTIS - A database of alternative medical literature. It indexes many peer-reviewed articles from several disciplines including chiropractic, osteopathy, homeopathy, and manual medicine. Available from Health Index (database builders) and major vendors. (This project used it via a subscription with OVID).
http://www.healthindex.com/MANTISPricing.html

PsycINFO - By the American Psychological Association. It is an abstract database of psychological and related disciplines literature. Subjects covered include: medicine, psychiatry, nursing, sociology, education, pharmacology, physiology, linguistics and other areas. (This project used it via a subscription with OVID).
http://www.apa.org/psycinfo/
